# Burkitt Lymphoma Presentation with Oropharyngeal Mass of Tonsillar Fossa: A Case Report

**DOI:** 10.5811/cpcem.42233

**Published:** 2025-08-13

**Authors:** Diormi A. Rosario, Stephanie Aronson, Jessica Zerzan

**Affiliations:** Maimonides Medical Center, Department of Pediatric Emergency Medicine, Brooklyn, New York

**Keywords:** Burkitt lymphoma, oropharyngeal mass, head and neck cancer, pediatric, lymphoma, oropharyngeal tumor

## Abstract

**Introduction:**

Burkitt lymphoma is a highly aggressive subtype of non-Hodgkin lymphoma with varied clinical presentation, including in some cases involvement of the intraoral cavity. Early recognition of this malignancy is critical, as it typically responds well to prompt and intensive treatment. In this case report, we present a rare manifestation of Burkitt lymphoma presenting as an oropharyngeal mass.

**Case Report:**

An eight-year-old male presented with tonsillar swelling and new-onset oral bleeding. A month earlier, he had been seen in the emergency department (ED) for similar swelling following a streptococcal infection. At that time, a needle aspiration for suspected peritonsillar abscess yielded no drainage, and he was treated with a week of clindamycin, resulting in brief symptom improvement. He subsequently developed difficulty breathing, a muffled voice, and oral bleeding, prompting a return to the ED. On evaluation, he was afebrile, well-appearing, and in no respiratory distress. Examination revealed significant left tonsillar swelling with uvular deviation but no active bleeding. Magnetic resonance imaging demonstrated a bulky left oropharyngeal mass with airway narrowing, raising suspicion for lymphoma. Laboratory results were unremarkable, and biopsy confirmed Burkitt lymphoma based on c-MYC positivity and the characteristic “starry sky” appearance, leading to the initiation of chemotherapy.

**Discussion:**

Burkitt lymphoma is a high-grade lymphoma with a large tumor burden and, thus, high risk for tumor lysis syndrome. Fortunately, Burkitt lymphoma has superior survival outcomes in pediatrics with a two-year survival rate estimated to be 89% and requiring minimal cycles of chemotherapy. This case underscores the diverse presentations of Burkitt lymphoma and the importance of including it in the differential for all pediatric neck masses, regardless of demographics.

## INTRODUCTION

Non-Hodgkin lymphoma is one of the most prevalent neoplasms seen worldwide.^1^ It can have many variants, including Burkitt lymphoma. Burkitt lymphoma is divided into three subgroups: sporadic, endemic, and immunodeficiency related.^2^ In the United States, the frequency of non-Hodgkin lymphoma in the pediatric population is 0.5–1.2 per 100,000.^3^ The presentation of Burkitt lymphoma usually involves the abdomen, mandible, or maxilla; however, in rare instances it can involve the oral cavity. This case depicts a rare presentation of Burkitt lymphoma, arising as a peritonsillar mass.

## CASE REPORT

An eight-year-old male presented with tonsillar swelling and new-onset oral bleeding. He had been seen in the emergency department (ED) one month earlier for similar symptoms and was treated with a seven-day course of clindamycin for suspected bacterial tonsillitis after a non-productive needle aspiration. His symptoms initially improved but later progressed to include difficulty breathing and a muffled voice. On the day of presentation, oral bleeding developed, prompting return to the ED. The family denied fever, drooling, dyspnea, or weight loss at that time.

**Image 1:**
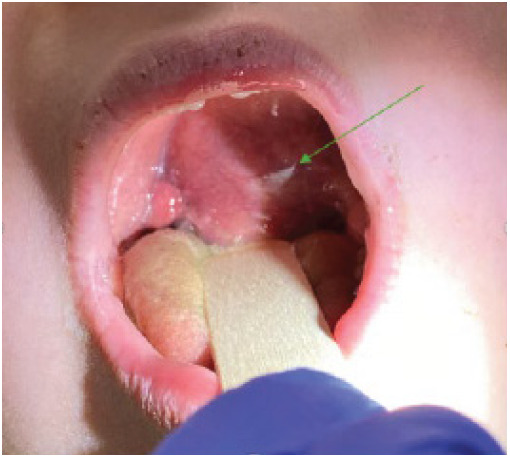
Left intraoral mass occluding part of the oral cavity (arrow).

Upon arrival to the ED the patient was afebrile with heart rate 101 beats per minute, respiratory rate 17 breaths per minute, blood pressure 121/63 millimeters of mercury, and oxygen saturation 98% on room air. On examination the patient was well-appearing, in no respiratory distress without stridor, and with marked left tonsillar swelling with uvular deviation, and no active bleeding (Image 1). The ear, nose, and throat (ENT) physicians were contacted, who recommended a magnetic resonance imaging (MRI) of the neck. The patient received a dose of ampicillin-sulbactam and was admitted with plans for biopsy with ENT. Labs were obtained, which included complete blood count, basic metabolic panel, magnesium, phosphorus, lactate dehydrogenase, uric acid, and venous blood gas; all were unremarkable.

**Image 2:**
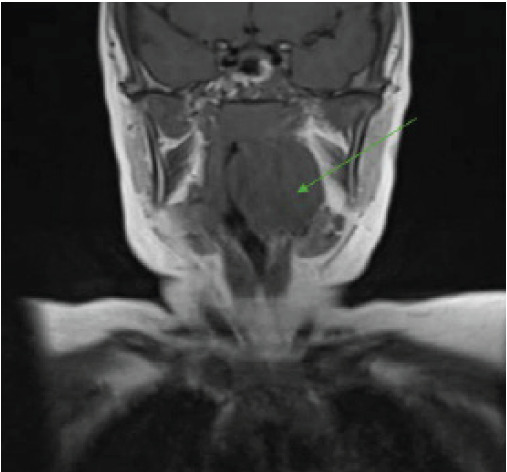
Magnetic resonance imaging coronal view depicting oropharyngeal mass (arrow).

**Image 3:**
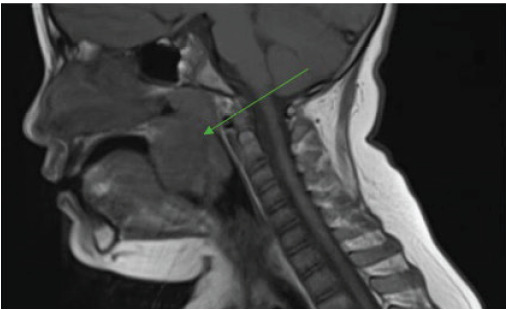
Magnetic resonance imaging sagittal view depicting oropharyngeal mass (arrow).

During the admission, the patient remained stable on room air. The MRI demonstrated a bulky well-circumscribed left oropharyngeal mass involving the tonsillar fossa, suspicious for lymphoma or other neoplasms, with narrowing of the oropharyngeal and nasopharyngeal airway (Images 2 and 3). Oncology was consulted, which recommended a biopsy. The ENT physician performed a biopsy of the left intraoral mass, debulking of mass, and endotracheal intubation. The biopsy was positive for c-MYC with “starry sky” appearance, all indicative of Burkitt lymphoma. The patient was then started on chemotherapy and was able to be extubated and discharged from the hospital.

*CPC-EM Capsule*

What do we already know about this clinical entity?

*Burkitt lymphoma is a rapidly growing B-cell tumor which often presents with abdominal pain of mandibular masses*.

What makes this presentation of disease reportable?

*Burkitt lymphoma is known to mimic many common pediatric conditions which can sometimes delay the diagnosis of such a rapid growing pathology*.

What is the major learning point?

*Burkitt lymphoma can present subtly, therefore persistent symptoms warrant escalation to rule out malignancy*.

How might this improve emergency medicine practice?

*This case helps heighten awareness to include maligmancy in differential diagnosis for persistent abdominal pain or systemic symptoms in children*.

## DISCUSSION

Burkitt lymphoma is not often on the differential diagnosis of an otherwise healthy child presenting with an oropharyngeal mass. As in our patient’s case, a child would be evaluated primarily for a peritonsillar abscess, peritonsillar cellulitis, retropharyngeal abscess, or infectious mononucleosis.^4^ While Epstein-Barr virus causes infectious mononucleosis, the association of Epstein-Barr virus with Burkitt lymphoma is not usually considered in the United States. Epstein-Barr virus is more likely to be associated with the endemic form of Burkitt lymphoma, which frequently develops in African children and adolescents.^5^ The sporadic form of Burkitt lymphoma, which has no geographic focality, is more often found in adult patients, often develops as an intra-abdominal mass rather than an oropharyngeal or peritonsillar mass.^6^ Our patient’s presentation was puzzling, in that he did have a muffled, “hot potato” voice in the setting of an oropharyngeal mass, seeming more like a peritonsillar abscess; however, he was otherwise well, afebrile, not in pain, and had no drainage on attempted incision and drainage by the ENT physician prior to ED presentation. Nonetheless, due to his demographics and presentation, Burkitt lymphoma was considered; however, it was low on our initial differential diagnosis.

Burkitt lymphoma is a high-grade lymphoma with a large tumor burden and, thus, high risk for tumor lysis syndrome. Tumor lysis syndrome occurs when tumor cells rapidly break down and intracellular contents are released into circulation, causing major electrolyte disturbances (hyperkalemia, hyperphosphatemia, hypocalcemia, and hyperuricemia). The electrolyte changes may cause an array of symptoms, ranging from muscle cramps and acute kidney injury to potentially fatal cardiac arrhythmias and seizures.^7^ Due to the tumor burden and high speed of growth, Burkit lymphoma patients are at high risk of tumor lysis syndrome and must be started on appropriate fluids and hypouricemic agents as soon as possible. Despite our patient’s fast progression of the tumor (from ENT visit to ED), he did not develop tumor lysis syndrome, once again lowering clinical suspicion for Burkitt lymphoma.

Fortunately, Burkitt lymphoma has superior survival outcomes in pediatrics. Recent studies have shown the two-year survival rate to be 89%, and most patients are treated with only four cycles of chemotherapy.^2^

## CONCLUSION

This case highlights the variability in initial presentation and serves as a reminder to include Burkitt lymphoma on the differential diagnosis for all pediatric neck masses regardless of the demographics.
